# Ethylene Response Factor 6 Is a Regulator of Reactive Oxygen Species Signaling in *Arabidopsis*

**DOI:** 10.1371/journal.pone.0070289

**Published:** 2013-08-05

**Authors:** Nasser Sewelam, Kemal Kazan, Skye R. Thomas-Hall, Brendan N. Kidd, John M. Manners, Peer M. Schenk

**Affiliations:** 1 Plant-Microbe Interactions Laboratory, School of Agriculture and Food Sciences, The University of Queensland, Brisbane, Queensland, Australia; 2 Commonwealth Scientific and Industrial Research Organization Plant Industry, Queensland Bioscience Precinct, Brisbane, Queensland, Australia; Michigan State University, United States of America

## Abstract

Reactive oxygen species (ROS) are produced in plant cells in response to diverse biotic and abiotic stresses as well as during normal growth and development. Although a large number of transcription factor (TF) genes are up- or down-regulated by ROS, currently very little is known about the functions of these TFs during oxidative stress. In this work, we examined the role of ERF6 (ETHYLENE RESPONSE FACTOR6), an AP2/ERF domain-containing TF, during oxidative stress responses in Arabidopsis. Mutant analyses showed that NADPH oxidase (RbohD) and calcium signaling are required for ROS-responsive expression of *ERF6*. *erf6* insertion mutant plants showed reduced growth and increased H_2_O_2_ and anthocyanin levels. Expression analyses of selected ROS-responsive genes during oxidative stress identified several differentially expressed genes in the *erf6* mutant. In particular, a number of ROS responsive genes, such as *ZAT12*, *HSF*s, *WRKY*s, *MAPK*s, *RBOH*s, *DHAR1*, *APX4*, and *CAT1* were more strongly induced by H_2_O_2_ in *erf6* plants than in wild-type. In contrast, *MDAR3*, *CAT3*, *VTC2* and *EX1* showed reduced expression levels in the *erf6* mutant. Taken together, our results indicate that ERF6 plays an important role as a positive antioxidant regulator during plant growth and in response to biotic and abiotic stresses.

## Introduction

Reactive oxygen species (ROS) are produced constantly during normal plant growth and development (e.g. during photosynthesis) and they also fulfill essential roles as highly specific signaling molecules under stress conditions. However, due to their highly toxic nature, ROS are also constantly scavenged by complex and redundant antioxidant defenses. Under various biotic and abiotic stress conditions such as high-light, drought, heat or pathogen attack, excessive amounts of ROS are produced and the balance between ROS production and degradation is disturbed, with potentially damaging consequences to cellular machinery [4], [14]. Given the importance of ROS as both damaging and signaling molecules, a better understanding of plant processes involved in ROS generation, signaling and scavenging is of significant importance in both basic plant biology and crop improvement.

In plants, ROS are produced through multiple pathways which include photosynthetic and respiratory electron transport chains, photorespiration, amine oxidases, cell wall-bound peroxidases, and membrane-bound NADPH oxidases (reviewed by Mittler et al., [43]). Membrane-bound NADPH oxidases also known as respiratory burst oxidase homologs (Rboh) are a group of enzymes that catalyze the production of superoxide radicals in both animals and plants (reviewed by Suzuki et al., [66]). Recent studies also show intimate links between ROS and plant hormones [43]. In stomatal guard cells, for instance, the plant hormone ABA activates ROS production through the NADPH oxidase RbohD and this leads to stomatal closure [21], [25]. Another study has shown that DELLA proteins with roles in GA-signaling regulate plant growth and stress tolerance through modulation of ROS levels [2]. Furthermore, other plant hormones such as auxin and plant defense hormones salicylic (SA) and jasmonic acid (JA) modulate the plant’s ROS status [43]. These studies suggest that plants expediently integrate signals from multiple endogenous and exogenous cues that lead to the modulation of cellular ROS levels.

Emerging evidence also indicates that both the level and sub-cellular location of ROS can induce specific cellular processes. For instance, ROS required for maintaining normal growth and development is produced at low levels and specifically where it is needed such as in root tip cells [28], [60]. In contrast, higher amounts of ROS produced under stress conditions can negatively affect plant growth. During challenge by an incompatible pathogen, ROS is specifically generated in the extra-cellular spaces of cells undergoing programmed cell death [68]. This hypersensitive-type (HR) response is genetically controlled by the plant and is often considered to be a useful evolutionary trait against the threat by biotrophic pathogens [62]. However, necrotrophic pathogens as part of their infection strategy, deliberately induce the production of ROS and cell death which facilitates subsequent tissue colonization [9], [67]. Similarly, under severe abiotic stress conditions, excessive amounts of ROS are generated as a result of cellular damage. Therefore, plants have also evolved mechanisms to protect themselves from the danger posed by ROS through various antioxidant defenses. Indeed, ROS coordinately activate the expression of genes encoding enzymes for ROS scavenging or synthesis of antioxidant enzymes or molecules required to counteract the potentially damaging effects of ROS. At least ten major cellular mechanisms involved in ROS removal are known (reviewed by Mittler [41]). These include several enzymatic mechanisms that involve the action of antioxidant enzymes such as superoxide dismutase (SOD), which converts O**^.−^**_2_ to H_2_O_2_, and catalases and peroxidases, which remove H_2_O_2_. The harmful effects of ROS can also be neutralized by non-enzymatic means through antioxidant molecules such as ascorbic acid, glutathione, carotenoids, and α-tocopherol. Furthermore, different ROS (such as superoxide radicals, H_2_O_2_ or singlet oxygen ^1^O_2_) produced in different subcellular compartments (*e.g*. plastids, mitochondria and peroxisomes) induce specific adaptive responses. For example, cytosolic H_2_O_2_ induces the expression of heat shock proteins during light stress [57]. In contrast, peroxisomal photorespiration-dependent H_2_O_2_ has a negative effect on the high-light stress induction of transcripts within the biosynthetic pathway for antioxidant anthocyanins [70].

Specific ROS sensors are not known; however, after perception, ROS signals are transmitted to downstream components by the action of secondary messengers such as G proteins, calcium ions (Ca^2+^), MAP-kinases and plant hormones [6], [26], [31], [40], [50], [59], [75]. Redox sensitive TFs activated by ROS then can stimulate the transcription of a large number of genes. Gadjev et al. [15] monitored the expression of the 1.500 transcription factors of Arabidopsis in response to different ROS, such as H_2_O_2_,**^.^**O**^−^**_2_, and singlet oxygen and found that ROS altered the expression of about one-third of all known TFs in Arabidopsis. In the study of Gadjev et al. [15], WRKYs, C_2_H_2_ zinc finger proteins and AP2/ERFs were found to be highly responsive to ROS. However, so far only few ROS-responsive TFs have been investigated functionally for their roles in oxidative stress signaling. For instance, members of the EAR-repression domain containing C_2_H_2_ zinc finger TFs have been linked to controlling ROS levels. Of these, ZAT12, which is required for cytosolic *ascorbate peroxidase1* (*APX1*) expression plays a central role in reactive oxygen signaling in Arabidopsis [9], [56]. Another member of this gene family, *ZAT10*, provides increased tolerance to ROS generated during photo-oxidative stress when over-expressed in transgenic plants [57]. Recently, JUB1, a ROS-responsive NAC TF regulating longevity in Arabidopsis, was shown to dampen intracellular H_2_O_2_ levels and to enhance tolerance to various abiotic stresses [73].

In this study, we investigated the potential functions of *ERF6*, a ROS-responsive AP2/ERF (APETALA2/ETHYLENE RESPONSE FACTOR) TF during oxidative stress. ERF6 is one out of 122 ERF TFs in Arabidopsis that belongs to group IX [46] which also comprises ERF1, ERF14 and ORA59 with well-demonstrated roles in plant innate immunity. ERF6 was also found to be induced by *Botrytis cinerea*, a necrotrophic pathogen, in Arabidopsis wild-type, *ein2* and NahG plants, but not in *coi1* [1], suggesting that ERF6 is dependent on *coi1*-mediated JA signaling. ERF6 is phosphorylated by MPK6 leading to defense gene expression and resistance against *B. cinerea* [38] and has also been shown to bind to another highly homologous member of this group, ERF5, with roles in the chitin-induced signaling network [64]. Double *erf5/erf6* mutants showed altered pathogen resistance [64] and dysfunctional induction of aliphatic glucosinolates by insects [37]. A recent study on the ERF6 protein has shown that it interacts with MPK6 to modulate oxidative gene expression [72]. In our study, *ERF6* showed a unique expression pattern as it was rapidly induced by ROS as well as pathogen, SA and cold stress. In contrast, *ERF6* was suppressed by water deficit and heat as well as by abscisic acid (ABA). Our results from the analysis of *erf6* knockout mutants suggest that ERF6 is required for controlled ROS production during plant growth, as well as biotic and abiotic stress signaling. By modulating the expression of genes encoding antioxidant enzymes, ERF6 alters the ROS level in plants which may then affect subsequent ROS-mediated signaling.

## Materials and Methods

### Plant Materials and Growth Conditions

All experiments in this study have been carried out with *Arabidopsis thaliana* ecotype Columbia (Col-0). The mutants used in this work were all SALK T-DNA insertion lines in Col-0 background. The *erf6* insertion line was SALK_087357. For soil-grown Arabidopsis plants, seeds were sown on soil and stratified at 4°C for 2 days before being transferred to a growth chamber at 24°C and 8 h photoperiod (150 µmol m**^−^** ^2^ s**^−^** ^1^). After ten days, seedlings were transplanted to new soil. At the age of 4–5 weeks, plants were treated or inoculated. Control plants were mock-treated. For further analysis, plant parts above the soil were collected. Three biological replicates (20 plants each) were used for each treatment. For plate-grown plants, Arabidopsis seeds were surface-sterilized (2 min in 70% ethanol then 15 min in 50% bleach and rinsed three times in distilled water) and sown on 1X MS (Murashige and Skoog) plates. Plates were kept at 4°C for 2 d, and then transferred to a growth cabinet at 24°C and 15 h photoperiod (150 µmol m**^−^** ^2^ s^_1^). 14-day-old plate-grown seedlings were subjected to different treatments. For further analysis the whole plants were collected. 50–60 healthy and similar seedlings from three different plates were used for RNA extraction and real-time RT-PCR. At the step of cDNA synthesis, three technical replicates were carried out. All treatments started at least 1 hour after lights switched on.

### Treatments

For chemical, high light and *Pseudomonas* treatments, 4 weeks-old soil-grown seedlings were used. For oxidative stress, plants were sprayed with freshly prepared 500 mM H_2_O_2_ or 30 µM paraquat (Sigma-Aldrich) solution (in water). Preliminary response experiments have shown that the relatively high concentration of H_2_O_2_ was necessary to ensure that sufficient H_2_O_2_ enters the cells; most likely because rapid degradation occurs in water and only a small proportion of the sprayed H_2_O_2_ is expected to penetrate through the waxy layers and cell walls of the leaves. Mock-treated plants were sprayed with water. For ABA and SA treatments, after dissolving in ethanol, a final concentration of 400 µM ABA or 4 mM SA in 1% ethanol was used for plant spraying [3]. Mock-treated plants were sprayed with 1% ethanol solution. The pH was adjusted to about 5 in hormone and mock treatments. For high light treatment, plants were transferred to a growth cabinet with a light intensity of 400 µmol photons.m**^−^** ^2^.s**^−^** ^1^. For *Pseudomonas* inoculations, *P. syringae* pv *tomato* strain DC3000 was grown in half-strength Luria-Bertani broth (LB) liquid medium containing the antibiotics kanamycin and rifampicin with final concentration of 50 µg/ml each. Bacteria from cultures with OD_600_ of 0.6 to 1 were collected by centrifugation at 3000×g for 10 min. The pellet was resuspended in sterile water to an OD_600_ of 0.2 (approximately 1×10^8^ colony-forming units/ml for *P. syringae,* DC3000). Using a 3-ml needle-less syringe, the abaxial (lower) sides of leaves from 4-week-old plants were gently pressure-infiltrated away from the midrib with freshly prepared bacterial cells. For the mock control, leaves were infiltrated with sterile water. Treated plants were covered with a transparent plastic dome to maintain high humidity. Heat, cold, water stresses and calcium channel blocker treatments were carried out on two-week-old MS plate-grown wild-type plants, as these treatments were easier to control and to compare under these conditions as opposed to soil-grown plants. Heat shock was conducted by heating plates in an incubator at 45°C (with light intensity of about 75 µmol photons m**^−^** ^2 ^s^_1^) for the indicated times. For cold treatment, plates were placed on ice and kept in a cold room (2°C). For water stress treatment, plants were removed carefully from the MS plates and placed on dry filter paper and left for the indicated time points. The mock-treated plants were placed on a filter paper wetted with distilled water. For calcium channel blocker treatments, seedlings were transferred carefully from MS-plates to Petri dishes containing filter paper wetted with distilled water and kept for 1 hour for recovery. For pre-treatment with the calcium channel blocker, Lanthanum (in the form of LaCl_3_) was added to a final concentration of 2 mM. After 1 hour, H_2_O_2_ was added to a final concentration of 50 mM. After five hours, seedlings were collected for further analysis.

### Real-time Quantitative RT-PCR

For RNA extraction plant samples were collected after the treatments, at the indicated time points, and were immediately immersed in liquid nitrogen and stored at -80°C. After grinding of twenty 4 weeks-old plants in liquid nitrogen to a fine powder, a representative sample of approximately 70 mg plant tissue was used for RNA extraction using the SV Total RNA Isolation System (Promega). RNA integrity was tested by gel electrophoresis and quantity measured by NanoDrop spectrophotometer (ND-1000 spectrophotometer). The same amount of RNA (from 1000 to 2000 ng) was used for cDNA synthesis in each experiment. SuperScript III reverse transcriptase (Invitrogen) was used for cDNA synthesis according to the supplier’s instructions. The Primer Express 2.0 software (Applied Biosystems) and DNA sequences, as templates, from the TAIR website (http://www.arabidopsis.org/) were used for primer design (Table 1). The primers were designed to amplify 100–150 bp close to the 3′ end of the gene. The specificity of the forward and reverse primers to the candidate gene was checked using the NCBI-BLAST website (http://www.ncbi.nlm.nih.gov/blast/Blast.cgi) and melting curve analysis following qRT-PCR. Primer efficiencies were incorporated into the data analysis and β-actin genes of Arabidopsis, *β-actin-2* (At3g18780), *β-actin-7* (At5g09810), and *β-actin-8* (At1g49240) primers were used as an internal control for normalization. Briefly, qRT-PCR was performed in optical 384-well plates using an ABI7900 HT Sequence Detection System (Applied Biosystems, Warrington, UK). Each reaction contained 6 µl of 2× SYBR Green Master Mix reagent (Applied Biosystems), 10 ng cDNA and forward and reverse gene specific primers at a concentration of 250 nM. The thermal profile comprised 95°C for 10 min followed by 45 cycles of 95°C for 15 s and 60°C for 1 min. Data were analyzed using SDS2.2 software (Applied Biosystems) and Microsoft Excel. Amplification plots were analyzed to provide cycle threshold values (Ct) using an Rn threshold of 0.3 for each primer pair-cDNA combination. PCR primer efficiency (E value) of each primer pair was calculated by linear regression analysis for each reaction. Absolute gene expression levels relative to *actin* reference genes was calculated for each cDNA sample using the equation: relative ratio gene/actin = (Egene–(Ct gene))/(E*actin*–(Ct *actin*)). Student’s t-test or two-way ANOVA (GraphPad Prism 5) was used to determine statistical significance.

**Table 1:** Real-time RT-PCR primer sequences.

Gene	Forward (5′ to 3′)	Reverse (5′ to 3′)
*RbohD*	TTCGAGTGGTTCAAGGGAATAATG	CGTACACACTCGTGCAATAATTGTG
*RbohB*	AGGAAATGTACTTTCACTTTACATGTCG	ATTGTAATGGTGAGACGTCAGAACAG
*EX1*	TCTGGTTTCCAGAGTTTCCTGC	GATGAAATCCTTATCCACCCTTCC
*OXI1*	CCAAGAGATTTTTGCTGCAAGAC	CCTTAACCCATTCCCCACTAGTATTATC
*MAPK6*	CATACCTGAACTCGTTGCACGAC	TCTGCTCCTCTGAGAGTGCATG
*MAPK3*	ACCAGTACCTTGCTAAATTGCACG	TCATCCAGAGGCTGTTGTTCG
*WRKY75*	CCAAAAGGCCGTCAAGAACAACAA	TGCTTCTTCACATTGCATCCTCCA
*WRKY40*	TGCGAGTTGAAGAAGATCCACCGA	TCCGAGAGCTTCTTGTTCTCAGCA
*ZAT12*	CCTAACAACGACGCTTTGTCG	GTCCCATCGGAAACTCCACTC
*HSFA4A*	CCAGGGCTTGCTTTGAACC	GGTTCATCGGGAAAGAACTCG
*HSF1*	TCCCAGATACCACAATTGACACG	TGAATGCCTCTGGAACATTCTTC
*MDAR1*	TTGGGTTCAAGGTGGTAAAGTGG	TCGAGCTTTGGCGACTTTAGC
*MDAR2*	GGAAAGTGGTTGGAGCATTTTTAG	CACTTCAAGGCTCTCAACAGAAGG
*MDAR3*	CTGAAGCCTGGTGAACTCGC	GGTCGGATTGACTTCGAGGTC
*DHAR1*	CTCTGACAAACCCCAGTGGTTC	CAACGATGACGTCGGAATCA
*APX4*	GCAACAGAGGCTGATCCAGAAG	CCAATCCAACAGCAATGAACTTATC
*CATALASE1*	CGTGAAGCGTTTTGTTGAAGC	CGAGTTGCTAGTTTCTGTCCCAG
*CATALASE2*	CTATCCGACCCACGCATCAC	TTCAGACGGCTTGCCAGC
*CATALASE3*	ACACCAGAGAGGGAAACTTTGATCT	TCCCATCACGGATGAAGAACA
*VTC2*	GATGGCAGCAAATTCAACTTCAC	GGCATGCAAGGGAAGAACTG
*HSP17*	TCATGAGGAGGTTTCGGTTGC	CTCTCCTGAACTTTCGGCACC
*PDF1.2*	CGCTGCTCTTGTTCTCTTTGC	GGGACGTAACAGATACAC
*ERD10*	AGCTCTTCTTCCTCTTCGAGTGATG	CCACTGTTTTCACATGATCTCCTTC

### Quantification of H_2_O_2_ and Anthocyanin Contents

H_2_O_2_ was assayed using the dye 2′,7′-dichlorofluorescein diacetate (H2DCFDA) according to the method of Joo et al. [21]. In parallel with each sample, catalase (300 unit/ml, Sigma) was added to subtract any unspecific H_2_O_2_ oxidation of the dye. The fluorescence was measured at 40 min after addition of the H2DCFDA dye using a fluorometer (Fluoroskan Ascent). Total anthocyanin content was measured according to the method of Rabino and Mancinelli [52]. Total pigment was extracted from 70 mg frozen plant tissue in 1 ml acidic (1% HCl) methanol. After centrifugation (5 min at 12,000 rpm in a microfuge) the supernatant was used for measuring the absorbance at 530 and 657 nm. Absorbance at 530 nm is specific for anthocyanin, but at 657 nm was used to compensate the background absorbance by chlorophyll. The equation A_530_–0.25A_657_ was applied for quantifying anthocyanin content.

### Monodehydroascorbate Reductase Assay

At 6 hours after H_2_O_2_ (500 mM) spraying, the 4-week-old plants were ground in liquid nitrogen. Total soluble protein was extracted from 0.1 g plant tissue in 1 ml cold (4°C) extraction buffer (1 mM ascorbate in 50 mM potassium phosphate buffer, pH 7.8). The homogenate was centrifuged at 4°C for 15.000 rpm. The supernatant was used immediately as enzyme extract. Total monodehydroascorbate reductase (MDAR) activity was assayed by following the decrease in NADPH via measuring the absorbance at 340 nm according to Hossain et al. [18]. Ascorbate oxidase (from *Cucurbita* sp. Sigma A 0157) was used to oxidize ascorbic acid producing monodehydroascorbate, which in turn was used to oxidize NADH by MDAR. The degree of NADH oxidation was taken as a measure of MDAR activity from plant tissue. The enzyme reaction (1 ml) contained 50 mM Tris-HCl buffer pH 7.6, 0.1 mM NADH (Sigma N 8129), 2 mM ascorbic acid, 0.2 units ascorbate oxidase and 50 µl enzyme extract. At 25°C, the reaction was initiated by addition of ascorbate oxidase. The enzyme activity was calculated using an extinction coefficient of 6.2 mM-1 cm-1 and normalized to the protein content. The protein concentration was measured according to Bradford [7].

### *erf6* Mutant Complementation

The wild-type *ERF6* gene was amplified from genomic DNA (extracted as described above) using the Expand High Fidelity System (Roche). A fragment of about 4000 bp (including, 2 kb upstream of the start codon for the promoter, 800 bp coding region, and 1200 bp downstream from the stop codon for the terminator) was amplified using the following primers, F: 5′-CGTTACACCAGAGTTGTGTG-3′ and R: 5′- GAGCTTACATGAGAGTCGAGC-3′. To check for errors during PCR, this *ERF6* fragment was cloned in the cloning vector pCR2.1 (TA Cloning Kit, Invitrogen). After verifying the correct sequence, the 4 kb fragment was cloned into the binary vector pGreenII0229 carrying the Basta herbicide resistance gene [18]. *Agrobacterium tumefaciens* strain (GV3103) was transformed with plasmid constructs (verified by sequencing and restriction enzyme analysis) through electroporation. Arabidopsis *erf6* mutant plants (SALK_087357) with many flowers and few pods were transformed by dipping the inflorescences into freshly prepared *Agrobacterium* (harboring the *ERF6* construct) solution (containing 5% sucrose and 0.03% Silwet L-77) for 10 seconds [32]. Using Basta screening, the homozygous complemented lines with single insertions were selected from the 3^rd^ generation. All measurements between *erf6* mutant and complemented *erf6* plants were referenced to wild-type plants. The expression of *ERF6* in the complemented lines was confirmed by qRT-PCR.

## Results

### *ERF6* Encodes a Reactive Oxygen Responsive Transcription Factor

**Figure 1:**
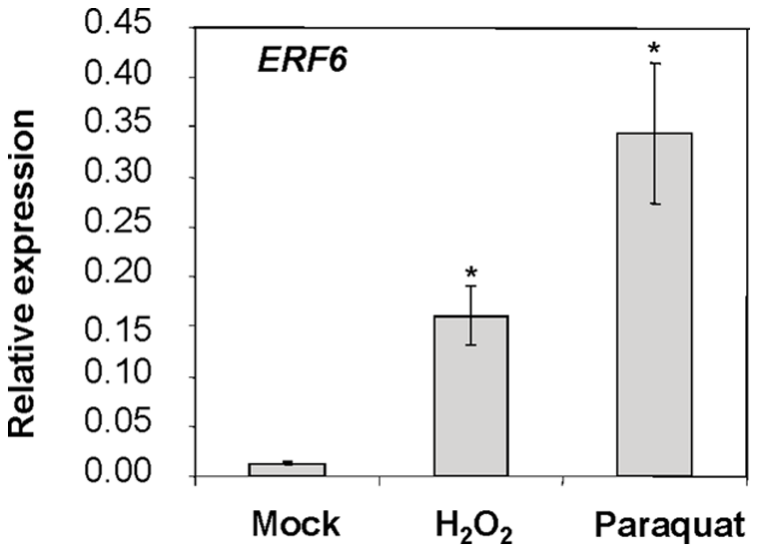
*ERF6* gene expression relative to actin genes analyzed by qRT-PCR after spraying 4-week-old soil grown Arabidopsis (WT, Col-0) plants with H_2_O_2_ (3h) or paraquat (2h). Three independent biological replicates (20 plants each) were used for each treatment. Error bars represent standard deviations. Asterisks indicate significant (*P*<0.05) differences in treated plants compared to mock-treated plants.

To identify transcriptional regulators of plant oxidative stress responsive gene expression, we examined the expression of several TF-encoding genes under oxidative stress imposed by the ROS (superoxide)-generating herbicide paraquat. The selected TFs included the members of the WRKY, AP2/ERF and C_2_H_2_ zinc finger TF gene families selected from the microarray dataset of Gadjev et al. [15]. Real-time quantitative RT-PCR (qRT-PCR) experiments showed that six TF genes, *ERF1*, *ERF2*, *ERF6*, *ZAT10*, *WRKY53* and *WRKY33*, were particularly early and strongly induced after treatment (Figure S1). Of these, *ERF6* was early and strongly induced as the expression of *ERF6* peaked at 2 and 3 hours, respectively, after paraquat and H_2_O_2_ treatment (Figure 1). The strong ROS responsiveness of *ERF6* indicated that this TF might be a regulator of ROS signaling in Arabidopsis. While the functions of the remaining five ROS-responsive TFs in plant hormone and stress signaling have been previously studied (ZAT10: [57]; ERF2: [36]; ERF1 and WRKY33: [20], [34]; WRKY53: [39], the function of ERF6 in ROS signaling is currently unknown. Therefore, in this study, we investigated the potential roles of ERF6 in oxidative stress signaling.

### ROS-dependent Expression of *ERF6* Requires Calcium and is Negatively Regulated by ZAT10 and MYC2

**Figure 2:**
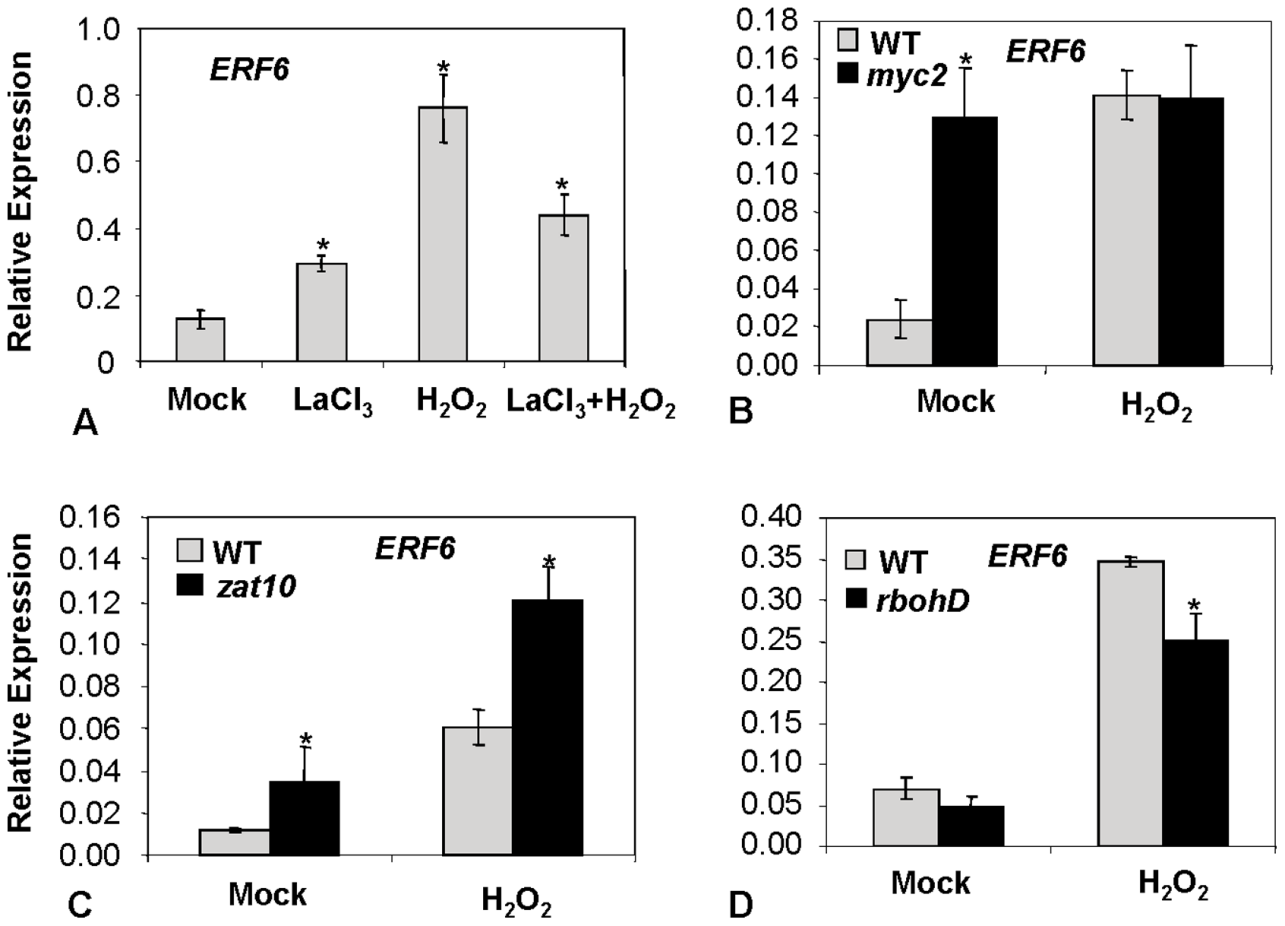
*ERF6* gene expression relative to actin genes analyzed by qRT-PCR. A) In wild-type (WT) Arabidopsis seedlings (grown on MS plates) pretreated with the calcium channel blocker La^3+^ followed by H_2_O_2_ treatment. LaCl_3_ was added at a final concentration of 2 mM, H_2_O_2_ was added at a final concentration of 50 mM. B, C and D). After spraying 4-week-old soil-grown Arabidopsis wild-type or mutant (*myc2*, *zat10* and *rbohD*, respectively) plants with H_2_O_2_ (6h). Three independent biological replicates (with 20 plants each) were used for each treatment. Error bars represent standard deviations. Asterisks indicate significant (*P*<0.05) differences in treated plants compared to mock-treated plants (A) or between mutant and WT (B-D).

Secondary messengers such as calcium are involved in mediating the transmission of ROS signals in both plant and animal cells [5]. Furthermore, Ca^2+^ is required for stimulation of the ROS-producing NADPH oxidase RbohD in plants [58], [66], the main NADPH oxidase involved in ROS production in leaves [68]. To study the involvement of calcium in ROS-mediated *ERF6* expression, wild-type Arabidopsis plants were treated with the calcium channel blocker Lanthanum chloride (LaCl_3_) prior to treatment with H_2_O_2_ and then *ERF6* expression was quantified. As shown in Figure 2A, pre-treatment of Arabidopsis plants with LaCl_3_ attenuated the induction of *ERF6* by H_2_O_2_, suggesting that Ca^2+^ signaling is required for the induction of *ERF6* by H_2_O_2_.

We also examined *ERF6* expression in the *zat10* and *myc2* mutants that have previously been shown to regulate ROS-related responses. The *zat10* mutant has been reported to show increased ROS accumulation [57] while the MYC2 TF was found to be a negative regulator of *ERF6* expression and the *myc2* mutant displayed increased ROS sensitivity [11]. In accordance with these previous findings, we found increased *ERF6* expression in the *zat10* mutant, particularly after treatment with H_2_O_2_, while basal *ERF6* transcript levels in untreated plants of the *myc2* mutant were elevated to levels equivalent to those observed in H_2_O_2_-treated wild-type plants but *myc2* plants were not further responsive to H_2_O_2_ treatment (Figure 2B and 2C).

### RbohD Contributes to ROS-responsive Expression of *ERF6* and *RBOHD* and *ERF6* are Co-regulated during Various Biotic and Abiotic Stresses

To determine whether *ERF6* expression is dependent on ROS produced via the NADPH oxidase RbohD, *ERF6* expression was measured in the *rbohD* mutant (SALK_083046) treated with H_2_O_2_. As shown in Figure 2D, ROS-responsive expression of *ERF6* was attenuated in the *rbohD* mutant background, suggesting that among other regulators RbohD contributes to the induction of *ERF6* during oxidative stress.

**Figure 3:**
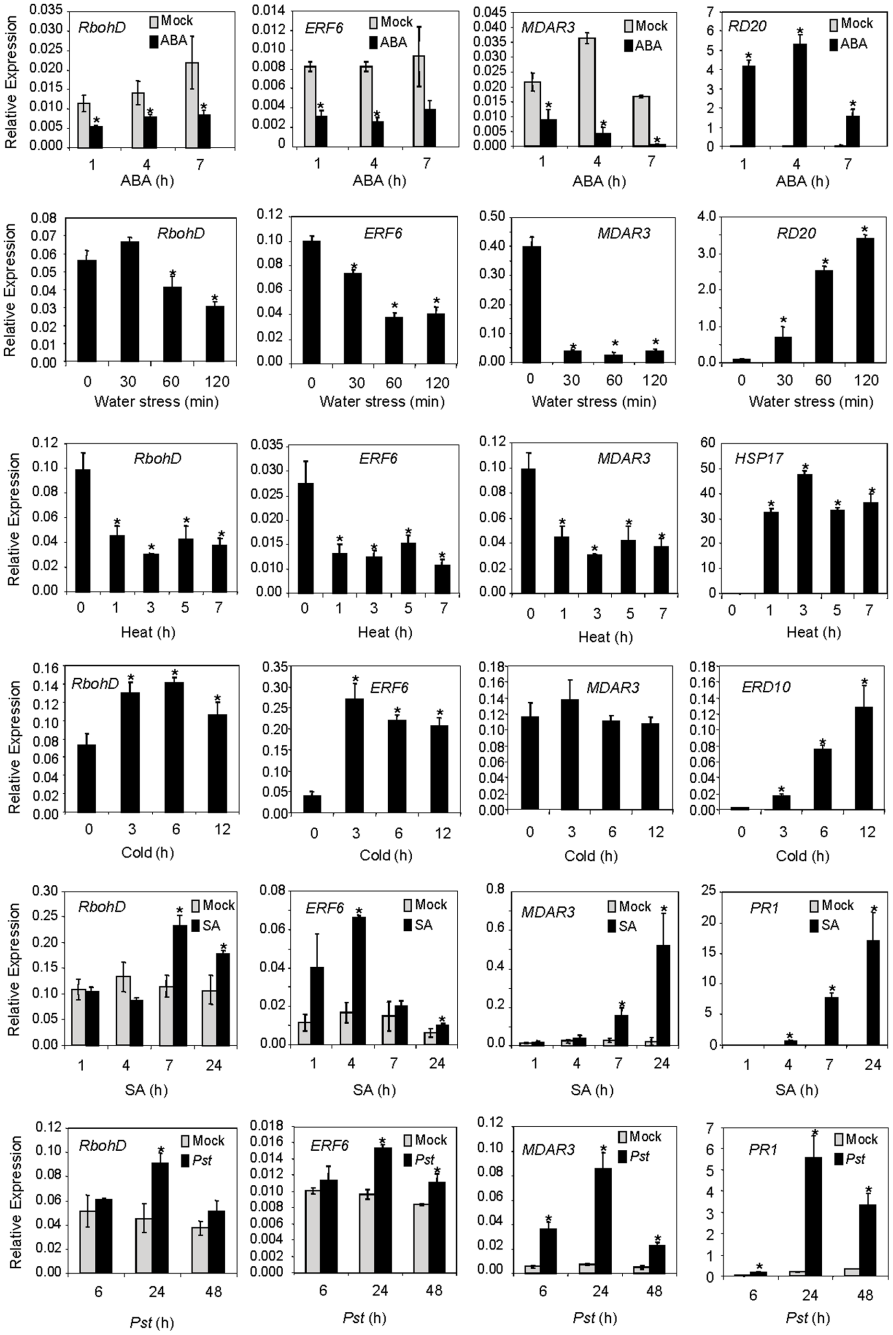
Gene expression patterns of *RbohD*, *ERF6* and *MDAR3* under different treatments by qRT-PCR. *RD20* is a marker gene for ABA and water stress treatments, *HSP17* is a marker for heat stress, *ERD10* is a marker for cold stress and *PR1* is a marker for SA and *Pseudomonas syringae* treatments. Three independent biological replicates (with 20 plants each) were used for each treatment. Error bars represent standard deviations. Asterisks indicate significant (*P*<0.05) differences in treated plants compared to mock-treated plants or to the time point before treatment.

To identify additional regulators of *ERF6* and to further explore the link between ERF6 and RbohD, we examined *RbohD* and *ERF6* expression in wild-type plants after treatment with heat, water stress, ABA, SA and inoculation with the bacterial pathogen *Pseudomonas syringae*. Expression of the SA- and pathogen inducible *PR1*, heat inducible *HSP17*, ABA and drought responsive *RD20,* and antioxidant biosynthesis *MDAR3* genes, was also analyzed. These experiments showed that biotic stress-related treatments, SA and *Pseudomonas syringae* inoculation, activated both *RbohD* and *ERF6* while abiotic heat and water stress treatments and ABA suppressed the expression of both genes (Figure 3). Interestingly, suppression of *RbohD* expression by abiotic stress treatments indicates that during abiotic stresses, plants might attempt to restrict excessive ROS accumulation through suppression of *RbohD* expression. These results indicate that *ERF6* is similarly regulated with the ROS production gene *RbohD* during diverse stress responses and we therefore hypothesized that ERF6 may play a role in the control of ROS levels in cells.

### *erf6* Mutant Plants Show Increased ROS Levels and Reduced Growth

**Figure 4:**
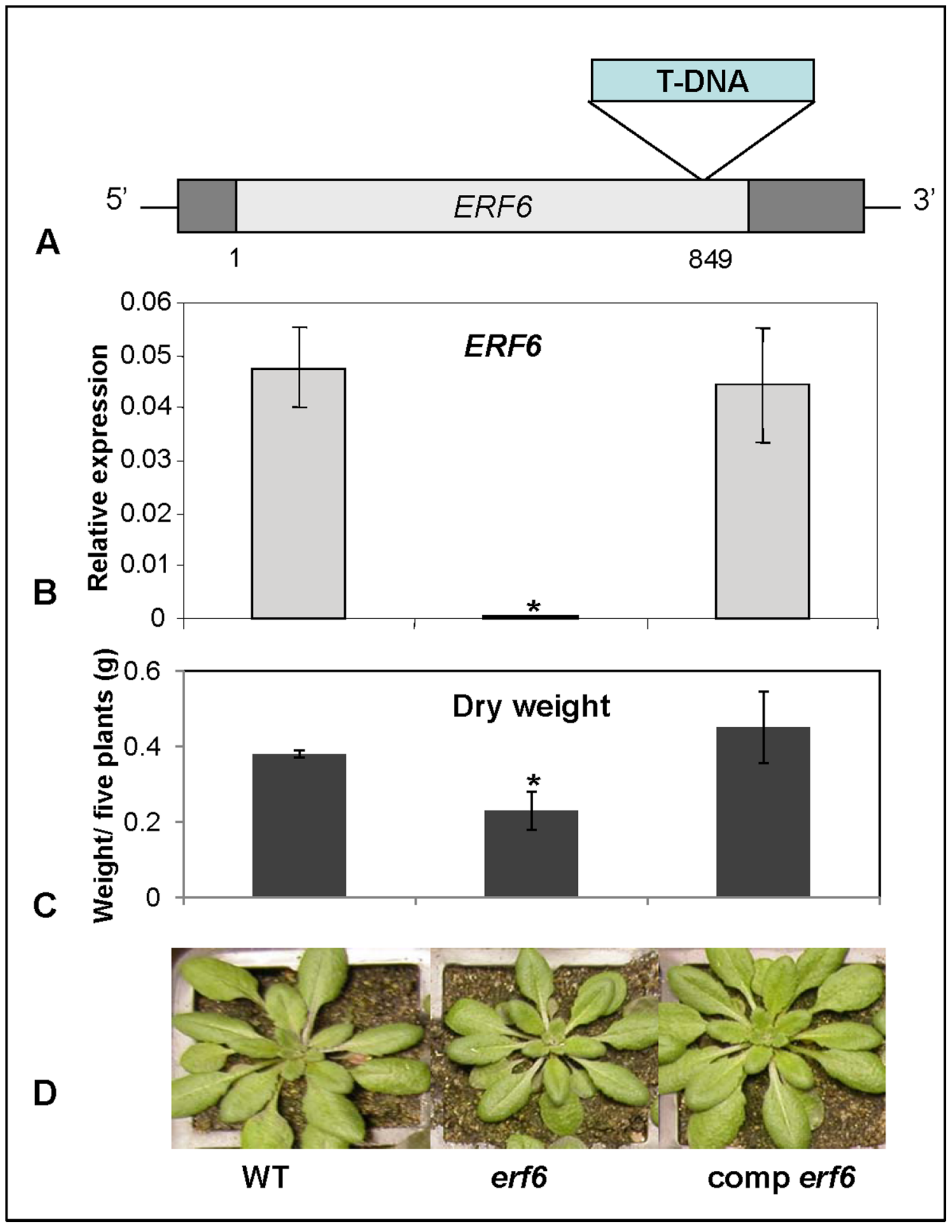
A) Schematic representation of the T-DNA insertion in the *ERF6* gene. Also shown are *ERF6* expression (B), Dry weights (C) and phenotype of whole soil-grown wild-type (WT), *erf6* mutant and complemented *erf6* mutant plants (comp *erf6*) grown under normal conditions. Three independent biological replicates (with 20 plants each) were used. Error bars represent standard deviations. Asterisks indicate significant (*P*<0.05) differences between *erf6* mutant compared to WT and complemented *erf6* plants.

To further investigate potential functions of ERF6 during oxidative stress, we examined a homozygous *erf6* T-DNA insertion line (SALK_087357) with a T-DNA inserted in the coding region of the *ERF6* gene (Figure 4A). There was no detectable *ERF6* mRNA in this line, confirming that this was a knockout line (Figure 4B). An independent study analyzing the nature of T-DNA insertions in Arabidopsis also confirmed that the *erf6* mutant analyzed here is a complete knockout for this gene [69]. However, this latter study did not report on any aspects of *ERF6* regulation or function.

**Figure 5:**
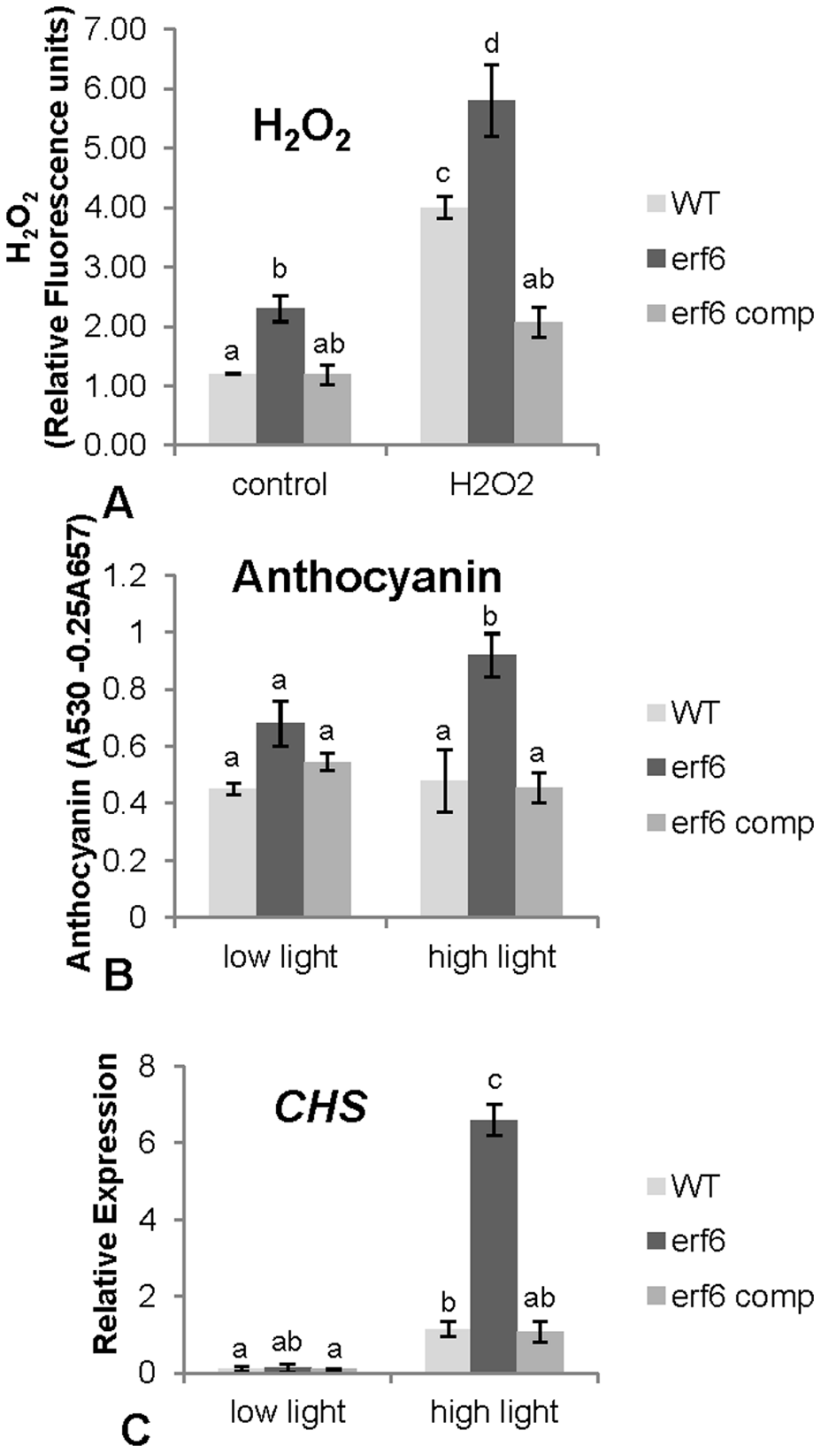
A) Hydrogen peroxide content of wild-type, *erf6* mutant and complemented *erf6* mutant plants was measured at 5 h after spraying 4-week-old soil-grown plants with H_2_O_2_. The content of H_2_O_2_ is expressed as relative fluorescent units. B) Anthocyanin content and C) *CHS* (*CHALCONE SYNTHASE*) expression in wild-type, *erf6* and complemented *erf6* plants was measured at 12 h after transferring plants from low light to high light conditions. Three independent biological replicates (20 plants each) were used for each treatment. Error bars represent standard deviations. Letters indicate significant differences determined by two-way ANOVA.

The *erf6* mutant plants grown under normal growth conditions described in Materials and Methods were smaller in size than the wild-type plants (Figure 4C–D). The reduced growth phenotype was particularly visible at the 6–8 leaf rosette stage. The dry-weight of *erf6* plants was only 73% of wild-type plants (Figure 4C). Transformation of *erf6* plants with a wild-type copy of *ERF6* including its native promoter restored wild-type expression levels of *ERF6*, and the complemented plants were phenotypically indistinguishable from wild-type (Figure 4B–D). The growth reduction phenotype of *erf6* plants suggested that the mutant plants may have been suffering from a stress. In many instances, plants defective in ROS scavenging or signaling contain increased ROS levels and display growth reduction [45]. To determine whether this was the case, the levels of H_2_O_2_, the most common and stable form of ROS, were measured in the *erf6* mutant plants. Results presented in Figure 5A show that *erf6* plants contained significantly higher levels of H_2_O_2_ than wild-type plants, both with (*P* = 0.008) and without (*P* = 0.003) exogenous H_2_O_2_ treatment. Therefore, the growth reduction of *erf6* might be at least partly ascribed to increased H_2_O_2_ levels found in the *erf6* mutant.

Under relatively high-light (400 µM.m**^−^** ^2^.s**^−^** ^1^) and long-day (16 h photoperiod) conditions, *erf6* mutant plants showed visibly increased anthocyanin pigmentation in their leaves compared to similarly grown wild-type plants. The quantification of anthocyanin showed that anthocyanin levels were significantly higher in *erf6* than in wild-type plants when grown either under high (*P* = 0.006) or light conditions (Figure 5B). The expression of *CHS*, a single-copy gene encoding for the chalcone synthase enzyme that catalyzes the biosynthesis of flavonoids, including anthocyanin [12] was also higher (*P* = 0.0001) in *erf6* under high light conditions than in wild-type plants (Figure 5C), most likely due to the oxidative stress imposed by increased H_2_O_2_ levels. In all three experiments, the complemented line restored the phenotype to equal or below the WT level suggesting that the *erf6* mutation is responsible for the increased phenotypes shown (Figure 5A–C).

To determine whether *erf6* plants show any altered sensitivity to exogenous ROS or plant hormones, wild-type and *erf6* seeds were germinated on MS medium containing H_2_O_2_, NaCl, SA, methyl jasmonate (MJ) or ABA and various growth characteristics such as germination rates and root elongation were scored. In these experiments, no discernible differences between wild-type and *erf6* plants were observed (Figure S2).

### Genes Regulated by ERF6 during Oxidative Stress

To identify genes that could be directly or indirectly regulated by ERF6 during oxidative stress, the expression of ROS- and plant defense-associated genes was analyzed in *erf6* and wild-type plants under oxidative stress conditions imposed by H_2_O_2_ treatment. These genes were chosen based on their differential expression or known role during plant defense and/or oxidative stress signaling [9,15,22,25,27,29,30,35,36,43,70,71,76; Table 1]. Seventeen genes showed significant differences (*P*<0.05) in expression between the *erf6* mutant and wild-type, suggesting that these are regulated by ERF6 during oxidative stress responses (Table 2). These differentially expressed genes in the *erf6* mutant plants included those associated with ROS biosynthesis (e.g. *RbohD*), signaling (e.g. *MAPK*s, *ZAT12*, and *WRKY*s), and scavenging (e.g. *DHAR1*, *APX4*, and *CAT1*). The genes that exhibited reduced induction in *erf6* plants relative to wild-type were *EX1* (*EXECUTER1*) encoding a plastid protein involved in singlet oxygen signaling [27], *MDAR3*, encoding a cytosolic mono-dehydroascorbate reductase enzyme involved in H_2_O_2_ detoxification, *CATALASE3* (*CAT3*) encoding an isoform of the catalase enzyme involved in ROS-detoxification and *VTC2* (*VITAMIN C DEFECTIVE 2*) encoding a GDP-L-galactose phosphorylase involved in the antioxidant vitamin C biosynthesis [30].

**Table 2:** Differential gene expression determined by qRT-PCR in *erf6* mutant compared to wild-type Arabidopsis plants under H_2_O_2_ treatment.

Functionalcategory	AGI Number	Gene	*erf6* to wild-type ratio*	*P* value**	Predicted/known location of gene product
**ROS Generation**					
	At5g47910	*RbohD*	4.56±1.82	0.039	Membrane
	At1g09090	*RbohB*	2.41±1.36	0.067	Membrane
**Signaling**					
	At4g33630	*EX1*	−6.78±1.63	0.059	Chloroplast
	At3g25250	*OXI1*	3.25±0.19	0.003	Unknown
	At2g43790	*MAPK6*	4.17±0.01	0.003	Various
	At3g45640	*MAPK3*	3.13±1.73	0.008	Various
	At5g13080	*WRKY75*	2.29±0.96	0.098	Nucleus
	At1g80840	*WRKY40*	2.68±0.66	0.017	Nucleus
	At5g59820	*ZAT12*	7.10±2.01	0.025	Nucleus
	At4g18880	*HSFA4A*	2.89±0.28	0.004	Nucleus
	At4g17750	*HSF1*	5.23±2.49	0.042	Nucleus
**Antioxidant and defense**					
	At3g52880	*MDAR1*	3.37±1.18	0.063	Peroxisome
	At5g03630	*MDAR2*	3.44±0.83	0.022	Cytosol
	At3g09940	*MDAR3*	−9.60±2.89	0.005	Cytosol
	At1g19570	*DHAR1*	6.41±1.46	0.019	Chloroplast
	At4g09010	*APX4*	2.47±0.78	0.013	Microsome
	At1g20630	*CATALASE1*	4.20±0.11	0.013	Various
	At4g35090	*CATALASE2*	3.39±1.04	0.057	Peroxisome
	At1g20620	*CATALASE3*	−12.83±2.77	0.028	Various
	At4g26850	*VTC2*	−8.77±3.12	0.011	Unknown
	At3g46230	*HSP17*	1.99±0.30	0.029	Unknown
	At5g44420	*PDF1.2*	5.02±0.67	0.004	Cell wall

The values represent the average of three biological replicates.

* Fold difference (2-fold or more) of gene expression in *erf6* plants compared to wild-type plants at 6 hours after H_2_O_2_ treatment ± SD. Negative signs indicate reduced expression in *erf6* compared to wild-type plants.

** P value, Student’s *t* test was used to calculate probabilities and to determine significant differences.

### ERF6 is Required for ROS-responsive Expression of *MDAR3*

**Figure 6:**
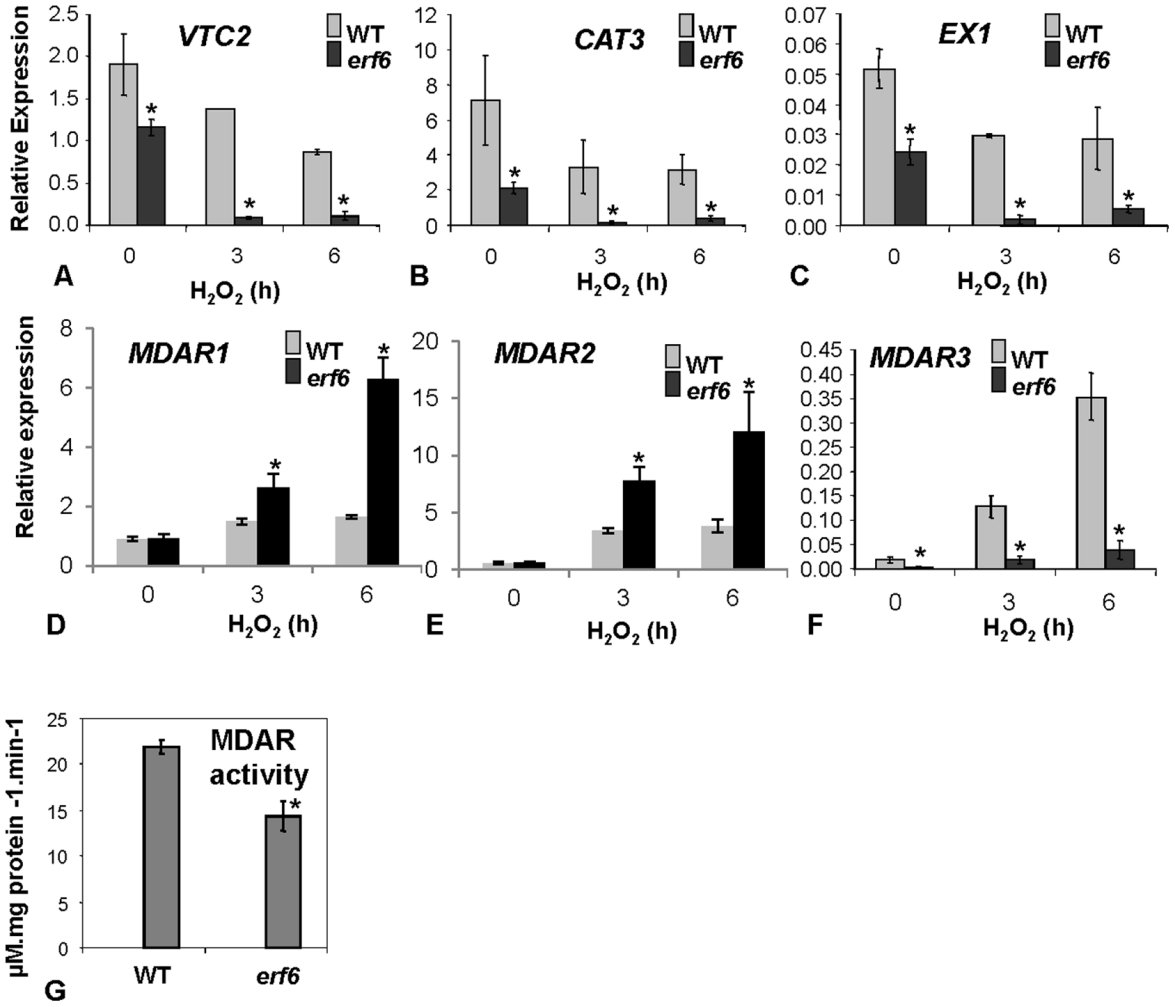
Gene expression levels of A) *VTC2* (*VITAMIN C DEFECTIVE2*), B) *CAT3* (*CATALASE3*), C) *EX1* (*EXECUTER1*), D) *MDAR1*, E) *MDAR2*, and F) *MDAR3* in WT and *erf6* plants after H_2_O_2_ treatment. Soil-grown 4-week-old Arabidopsis seedlings were either sprayed with H_2_O_2_ or with distilled water (control). The presented data for *MDAR1* and *MDAR2* represent the time point 6 h. G) Total MDAR activity in H_2_O_2_-treated wild-type (WT) and *erf6* plants. Enzyme activity was measured after 6 h from spraying plants with H_2_O_2_. Three biological replicates (20 plants each) were used for each treatment. Error bars represent standard deviations. Asterisks indicate significant (*P*<0.05) differences between *erf6* mutant and WT plants.

The stronger induction of ROS-responsive genes by H_2_O_2_ in the *erf6* mutant background could be due to the response of these genes to increased ROS levels in *erf6* plants but not due to a direct repressive effect imposed on these genes by ERF6. Therefore, we next focused on the genes that showed reduced induction by ROS in *erf6* plants as these genes could possibly be directly regulated by ERF6. In separate time-course experiments, we analyzed the expressions from *MDAR3*, *CAT3*, *VTC2* and *EX1* that showed reduced expression in the *erf6* mutant relative to wild-type plants (Figure 6). Interestingly, of these four genes, *EX1*, *CAT3* and *VTC2* were down-regulated in response to H_2_O_2_ treatment in both wild-type and the *erf6* mutant. However, expression levels of these genes in the H_2_O_2_-treated *erf6* mutant were lower than those in wild-type plants (Figure 6). These results suggest that similarly to the genes that showed up-regulation in the *erf6* plants, down-regulation of these three genes might simply be due to response to increased ROS levels in the *erf6* mutant. However, we noted that *MDAR3* was the only gene whose expression was induced by H_2_O_2_ in wild-type but not in *erf6* (Figure 6F). This suggests that ERF6 is required for ROS-responsive up-regulation of *MDAR3*. To determine whether *MDAR3* and *ERF6* are generally co-regulated in response to diverse biotic and abiotic stress conditions, we examined *MDAR3* expression in wild-type plants treated with biotic- or abiotic stress-related treatments. Remarkably, these experiments showed that similar to *RbohD* and *ERF6*, *MDAR3* was up-regulated in response to SA and *P. syringae* but down-regulated in response to heat and water stress treatments (Figure 3). Therefore, it is possible that *RbohD*, *ERF6* and *MDAR3* are all part of the same ROS-responsive regulon.

In contrast to *MDAR3*, *MDAR1* and *MDAR2* were induced more strongly by H_2_O_2_ in *erf6* than in wild-type plants (Table 2 and Figures 6D–6F). To determine what effect, if any, the differential regulation of different MDAR genes would have on overall MDAR levels, the total MDAR enzyme activity in crude soluble extracts of H_2_O_2_-treated wild-type and *erf6* plants was measured. Results from these experiments showed that total MDAR activity was less in the *erf6* mutant than in wild-type plants (Figure 6G). To examine to which extend the down-regulation of *MDAR3* in *erf6* plants is responsible for the phenotype of *erf6* plants, two homozygous T-DNA insertion lines of *MDAR3* (SALK_076335 and SALK_151778) were analyzed under a variety of growth conditions. However, *mdar3* mutant plants did not show any altered growth phenotype (data not shown). This result suggests that the increased H_2_O_2_ levels found in the *erf6* mutant probably resulted from the compromised expression of multiple antioxidant genes and thus the individual knockouts of these genes is unlikely to produce a phenotype similar to that seen in the *erf6* mutant.

## Discussion

Currently, a major gap exists in our understanding of how ROS induce large-scale and coordinated changes in expression from many genes. So far, only a few TFs have been found to be involved in regulating ROS-responsive gene expression. In this study, we investigated the role of ERF6 during oxidative stress. The ERF TF family is characterized by a single AP2/ERF DNA binding domain and comprises 122 members in 12 groups, representing one of the largest TF gene families in Arabidopsis [54]. Most genes in the ERF TF family are highly responsive to biotic and abiotic stresses (reviewed by Riechmann and Meyerowitz [55], Nakano et al. [46]) and at least some members of this family mediate responses to pathogen infection with roles in plant innate immunity, such as ERF1, ERF2, ERF4, ERF14 and ORA59 [36], [46] and abiotic stresses such as dehydration, salt and cold stress [13], [46], [49], [65]. Another member of this gene family, *RRTF1*, was found to play a major role in the adjustment of Arabidopsis leaves to high light stress [23].

In this study, we found that *ERF6* strongly responds to oxidative stress conditions imposed by either super-oxide-generating herbicide paraquat or H_2_O_2_ (Figure 1). Our exploration for upstream regulators of ROS-responsive expression of *ERF6* also identified calcium ions, as ROS-responsive expression of *ERF6* was attenuated in the presence of a calcium ion channel blocker (Figure 2). Similar to our results, several previous studies implicated ERF6 in plant stress responses. For instance, a study analyzing publicly-available microarray data from ROS treatments identified *ERF6* as one of the highly induced TFs by ROS [15]. Another similar study by Ma and Bohnert [35] has classified *ERF6* as a common stress responsive gene in Arabidopsis. In addition, *ERF6* was instantly induced in the *flu* mutants following the release of singlet oxygen [8]. *ERF6* was also responsive to bacterial and fungal elicitors such as flagellin [47] and chitin [29] as well as fungal pathogens *Alternaria brassicicola* [36] and *Botrytis cinerea* [1].

The strong induction profile of *ERF6* in response to ROS suggested an important role for ERF6 in ROS signaling. The *erf6* mutant also showed increased ROS levels and reduced growth as well as other stress-associated phenotypes such as increased accumulation of anthocyanin, particularly under high light intensities (Figure 5). This phenotype is consistent with an independent *erf6* mutant that was recently reported to show growth retardation and higher sensitivity to photodamage [72]. These results suggest that ERF6 is possibly either a negative regulator of ROS production or a positive regulator of ROS detoxification. However, *erf6* seedlings and wild-type plants exposed to ROS or ROS-producing stress conditions in plate assays were equally affected (Figure S2), suggesting that ERF6 is a regulator of chronic but not rapid ROS accumulation imposed by these stress factors. Consistent with a regulatory role of ERF6, a number of ROS-responsive genes showed altered expression in the *erf6* mutant. Among the genes that showed higher expression in the H_2_O_2_-treated *erf6* mutant compared to wild-type plants are the C_2_H_2_ zinc finger TF *ZAT12* (Table 2). Previously, a role for ZAT12 as a positive regulator of oxidative stress responsive gene expression has been reported [9]. Similarly, *MAPK3* and *MAPK6* and *OXI1* were differentially expressed in the *erf6* mutants (Table 2). MAPK3 and MAPK6 are involved in a variety of stress responses during oxidative stress including plant defense [17], [27] and have recently been shown to phosphorylate ERF6 *in vitro* [64]. It should be noted though that these kinases are mostly post-translationally regulated [27] and the altered transcript abundance may not be needed to have an effect on activity. Recent studies confirm the role of ERF6 in MPK3/MPK6-mediated plant defense responses [38], [44], [64], [72]. ERF6 when phosphorylated by MPK3 acts as a positive regulator for defense responses against necrotrophic pathogens [38], and binds as a MPK6/ERF6 protein complex to the GCC box [72]. OXI1 kinase functions upstream from MAP-kinase signaling pathways and is required for full activation of the MAP-kinases during oxidative burst [53]. *ERF6* expression was also reported to be up-regulated by transgenic expression of the activated MKK9 kinase, which is known to be an upstream activator of MPK3 and MPK6 kinases [74]. Also RbohD, a key factor in ROS production in Arabidopsis leaves [40], showed stronger expression in the *erf6* mutant than in wild-type plants. Although further analyses are required to determine whether ERF6 is involved in regulating other genes, these findings suggest an important role for ERF6 in cell signaling in Arabidopsis.

Interestingly, the genes encoding different isoforms of the same antioxidant enzymes showed differential expression in the *erf6* mutant. For example, *CAT1* and *CAT2* showed up-regulation while *CAT3* showed reduced expression in the *erf6* mutant relative to wild-type plants (Table 2). Similarly, *MDAR1* and *MDAR2* showed up-regulation while *MDAR3* showed down-regulation in the *erf6* mutant (Figure 6). It is possible that these genes might simply be responding to the increased H_2_O_2_ levels found in the *erf6* mutant. Unexpectedly, we also identified some antioxidant genes such as *VTC2*, and *CAT3*, whose expression was down-regulated by H_2_O_2_ in the wild-type but even more so in the *erf6* mutant plants (Figure 6). The biological significance of the suppression of these antioxidant genes by H_2_O_2_ is not clear but at least *VTC2* and *CAT3* seem to be responding to the increased ROS levels in the *erf6* mutant. It was proposed that for the operation of ROS-mediated signaling, down-regulation of certain antioxidant components might be necessary [14]. So, it is possible that *VTC2* and *CAT3* encode two such antioxidant components. However, *MDAR3* is an exception to this. Despite strong ROS responsiveness of *MDAR3* in wild-type plants, this gene could not be induced in the *erf6* mutant (Figure 6), suggesting that ERF6 is required for ROS-responsive up-regulation of *MDAR3,* but not *MDAR1* and *MDAR2*. Despite differential expression of different MDAR genes in the *erf6* mutant, the *erf6* mutant had reduced total MDAR activity. This finding suggests that the overall contribution of different MDAR genes to the final MDAR activity may be different. However, *mdar3* mutants did not display any *erf6*-like growth phenotypes. Therefore, it is likely that ERF6 controls other genes, in addition to *MDAR3*, which altogether contribute to the growth reduction of *erf6* plants. Furthermore, the effect of knocking out *MDAR3* might be redundantly masked by other members of the *MDAR* family or other antioxidant genes. A network comprising 152 antioxidant genes, including five different MDARs, is potentially involved in controlling the level of ROS in Arabidopsis [42].

The finding that most of the ROS-responsive genes analyzed showed up-regulation in the *erf6* mutant might indicate that ERF6 is a negative regulator of ROS-responsive gene expression. However, ERF6 does not contain the EAR (ERF-associated Amphiphilic Repression) domains typically found in the repressor type ERFs and C_2_H_2_ zinc-finger proteins (reviewed by Kazan [22]). Also, because AP2/ERF TFs bind to the conserved GCC-box found in the promoters of their target genes, we analyzed the promoters of genes showing differential expression in the *erf6* mutant for possible enrichment of the GCC-box motifs. The selected gene set examined for differential expression was relatively small and there was no obvious enrichment of the GCC-box or any other known conserved sequence element in the promoters of genes differentially regulated in the *erf6* mutant. The recent study by Wang et al. [72] has confirmed that the MPK6/ERF6 protein complex binds to a GCC box that was also predicted to present a ROS-responsive cis-acting element. Altogether, these observations reiterate the view that the ROS-responsive genes that show increased expression in the *erf6* mutant, may not all be directly regulated by ERF6, but are merely responding to the high H_2_O_2_ levels found in *erf6* plants.

### *ERF6* is Induced by Elevated ROS during Biotic or Abiotic Stress but also in a ROS-independent Manner when a Reduction of ROS is Required

Taking our data and the publically available expression data together, *ERF6* is induced during oxidative (H_2_O_2_, ^1^O_2_, O_3_, paraquat, UV-B), osmotic (NaCl, mannitol) and cold stress, as well as by necrotrophic pathogens (*B. cinerea*, *A. brassicicola*), coronatin-producing pathogen (*P. syringae*), pathogen elicitors (Flg22, hrpZ, cellulase, chitin), plant hormones (SA, MJ, IAA) and during early root development (Figure S3). In contrast, *ERF6* is repressed by heat and water stress, ABA, Cd, Avr, biotrophic powdery mildew (*Erysiphe orontii*) and by hemibiotrophic *Fusarium oxysporum*. The wide range of treatments that induce or repress *ERF6* expression all are linked to altered ROS levels in the plant. Given the proposed function of ERF6 as an antioxidant regulator, this suggests that many of these treatments (e.g. SA, *P. syringae*) might induce *ERF6* via the elevated ROS levels that they cause, either by cell/organelle damage or by active ROS production (e.g via RbohD). On the other hand, treatments that induce *ERF6* to actively achieve a reduction in ROS levels may do this via a ROS-independent *ERF6* induction. Examples of this are induction by MJ and necrotrophic pathogens (*B. cinerea*, *A. brassicicola*). Other abiotic stresses, such as wounding, Cd, heat and water stress result in elevated levels of ABA which also plays an important role in regulating stomatal opening and closure. The fact that *ERF6* and *RbohD* gene expression data were coordinated in our experiments (both were induced for oxidative and cold stress, but suppressed by heat and water stress) suggests that *ERF6* induction is either correlated to specific sources of ROS or fine-tuned by an interplay of ABA, RbohD and other factors influencing ROS levels (Figure 3; Figure S3). Despite *ERF6* and *RbohD* being co-expressed under defined short-term treatments, an increased basal *RbohD* transcript abundance was measured in the *erf6* mutant background (Table 2). It is possible that this may simply have occurred because of the increased accumulation of higher H_2_O_2_ levels in the mutant plants (Figure 5A), as higher oxidative stress has been shown to induce *RbohD* ([40]; Figure S3).

Although both biotic and abiotic stress leads to ROS generation in plant cells, the mechanism, perception and signaling of ROS produced in response to each of these stresses might be substantially different. In contrast to the pathogen sensing and recognition at the plant cell surface, abiotic stresses are sensed mainly through their damaging effects on living cells [4], [63]. Many reports suggested that physiological functions of cellular organelles, e.g. chloroplasts and mitochondria, are impaired when subjected to abiotic stresses such as drought [48], heat [33], salinity [75] or cold [62]. In these circumstances, ROS are produced as an inevitable consequence of cell damage. In contrast, ROS produced upon successful recognition of an incompatible pathogen depends largely on the “active” generation of ROS mainly produced by plasma membrane-bound NADPH oxidase enzymes [16], [67], [68]. Therefore, as a part of a negative feed-back loop, it is possible that during abiotic stress responses, the plant down-regulates RbohD via ABA (Figure 3; Figure S3) to avoid further generation of ROS.

Previous research has shown that ABA-mediated generation of H_2_O_2_ by RbohD in the stomatal guard cells plays an important role in the regulation of stomatal closure [21], [24], [25]. Contrary to this, our experiments showed that ABA suppresses *RbohD* expression and this was consistent in independent experiments. Similar to our results, other studies such as publicly available microarray data in Genevestigator [76] and by Wang et al. [71] showed that ABA suppresses *RbohD* expression. It should be noted, however, that we examined *RbohD* expression in ABA-treated whole leaves, whereas Kwak et al. [25] studied the expression of *RbohD* in stomatal guard cells. Therefore, ABA’s activation of *RbohD* expression is probably limited to guard cells. In fact, so far no study has shown that oxidative stress leads to stomatal closure or that ABA treatment causes oxidative stress (reviewed by Foyer et al. [14]). In conclusion, this study shows that *ERF6* plays an important role during oxidative stress signaling and is required for expression of antioxidant genes. Two recent studies also report that ERF6 plays a role as a positive regulator during JA/ET-mediated defense against *Botrytis cinerea* [44] and in chitin-mediated innate immune responses [64]. Taken together, this demonstrates that ERF6-mediated oxidative stress signaling is intimately linked to pathogen defense signaling, possibly via the action of ROS. Future studies will reveal further insights into plant ROS signaling from the study of other transcriptional regulators. A good candidate could be ERF5, the closest homolog to ERF6. ERF5 was recently shown to bind to ERF6 and both TFs act redundantly in JA/ET defense against *B. cinerea* [44], [64].

## Supporting Information

Figure S1 **Expression of selected transcription factor-encoding Arabidopsis genes analyzed by qRT-PCR after paraquat treatment compared to mock-treated plants.** Shown are data from three biological replicates (20 plants each) of 4 weeks-old soil-grown Arabidopsis (WT, Col-0) seedlings that were either sprayed with 30 µM paraquat or with distilled water (control). Error bars represent standard deviations. All expression levels from treated plants are significantly (*P*<0.05) different compared to those in mock-treated plants.
(TIF)

Figure S2 **Phenotypes of wild-type (Col-0) and** ***erf6*** **Arabidopsis seedlings on MS medium containing H_2_O_2_, NaCl, SA, MJ or ABA.** No discernible differences between wild-type and *erf6* plants were observed.
(TIF)

Figure S3 **A simplified model proposing the regulatory role of ERF6 in ROS signaling in Arabidopsis.** The model combines gene expression data from this study (treatments in bold letters; solid arrows) together with published results [36,37,38,44,64,69,72,76; dashed arrows] and proposes that transcriptional regulation of *ERF6* is mostly controlled by ROS levels in plant cells and then leads to a reduction of oxidative stress via anti-oxidant defenses. Cellular ROS levels are influenced by a number of factors, for example various abiotic stresses, NADPH oxidase action and anti-oxidant defenses. Thicker arrows may show the preferred signaling routes of various abiotic stresses that can lead to induction of *RbohD* and *ERF6* for oxidative and cold stress, but suppression by heat and water stress (see Figure 3). In addition, biotic stress caused by successful necrotrophic pathogens may increase ROS levels while typical defense actions against biotrophic pathogens and their elicitors (e.g. Avr) may stimulate ROS production via NADPH oxidase RbohD. Recent experimentation at the protein level has confirmed the role of ERF6 in modulation of cellular oxidative function [72].
(TIF)
