# Supplementary figures and images for: Ethylene Response Factor 6 Is a Regulator of Reactive Oxygen Species Signaling in Arabidopsis
Source: PLoS One. 2013 Aug 5;8(8):e70289. doi: 10.1371/journal.pone.0070289 (PMC3734174; doi:10.1371/journal.pone.0070289)

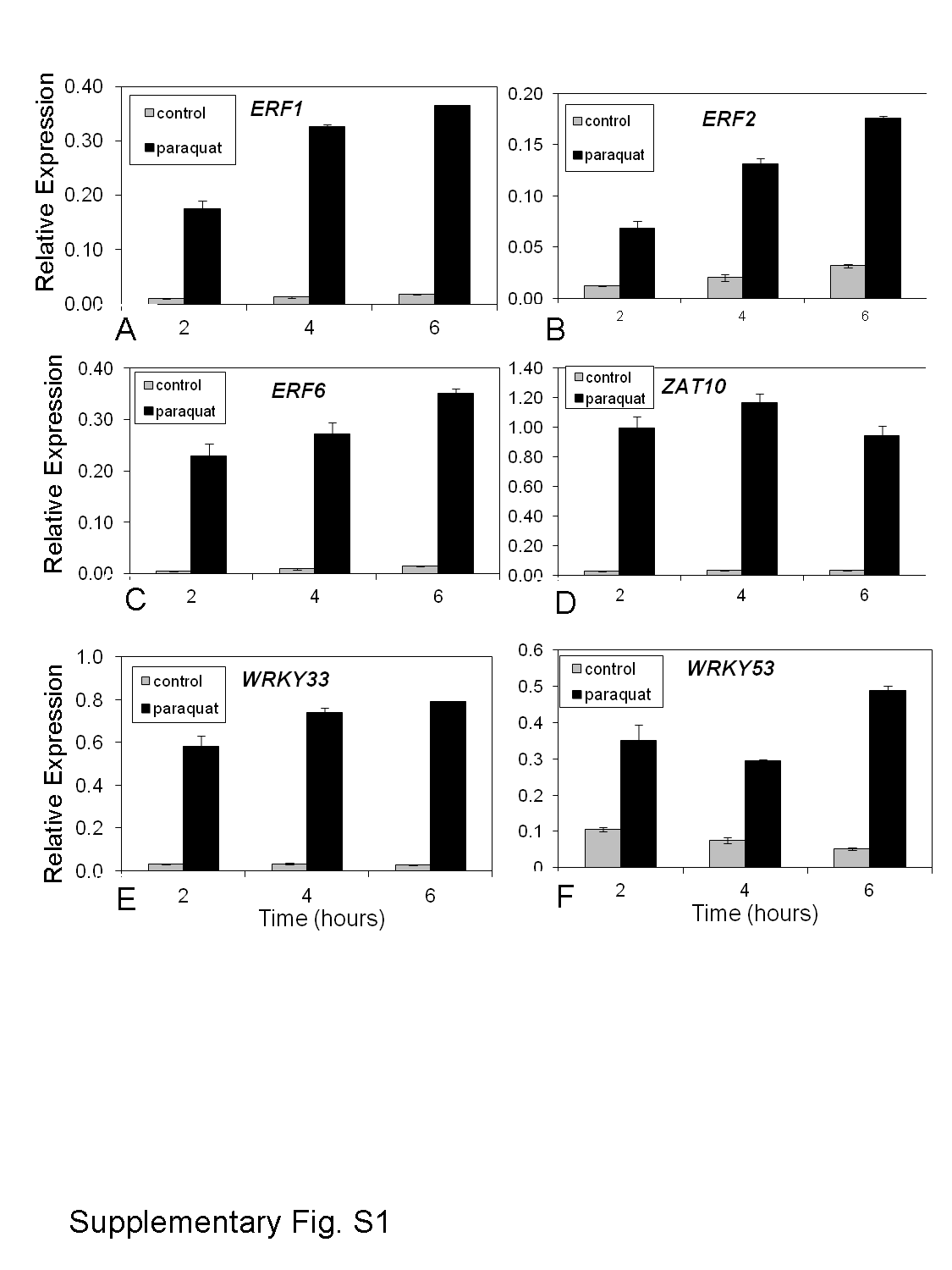

Supplement: Figure S1 — Expression of selected transcription factor-encoding Arabidopsis genes analyzed by qRT-PCR after paraquat treatment compared to mock-treated plants. Shown are data from three biological replicates (20 plants each) of 4 weeks-old soil-grown Arabidopsis (WT, Col-0) seedlings that were either sprayed with 30 µM paraquat or with distilled water (control). Error bars represent standard deviations. All expression levels from treated plants are significantly (P<0.05) different compared to those in mock-treated plants. (TIF) [file pone.0070289.s001.tif]

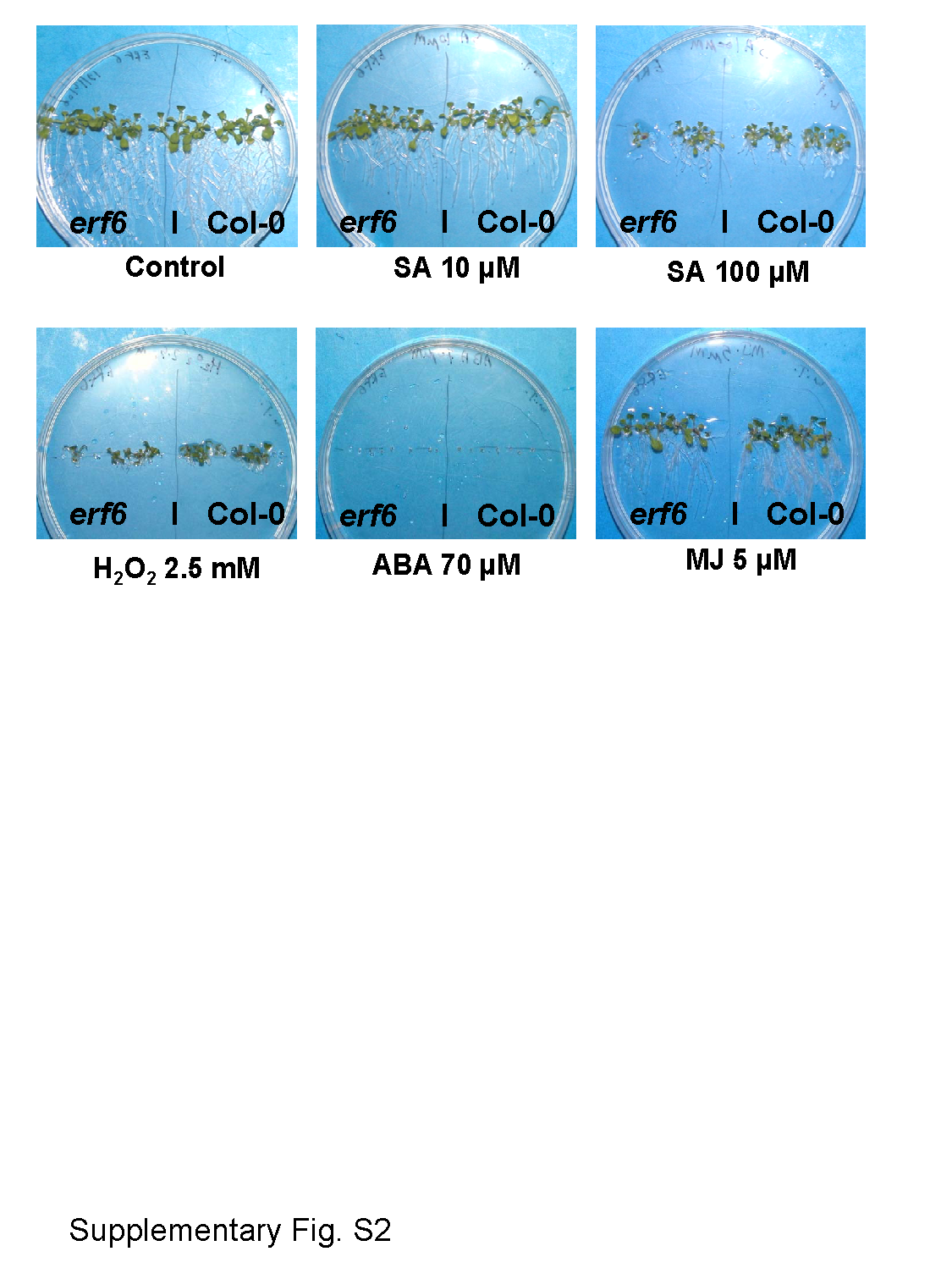

Supplement: Figure S2 — Phenotypes of wild-type (Col-0) and erf6 Arabidopsis seedlings on MS medium containing H2O2, NaCl, SA, MJ or ABA. No discernible differences between wild-type and erf6 plants were observed. (TIF) [file pone.0070289.s002.tif]

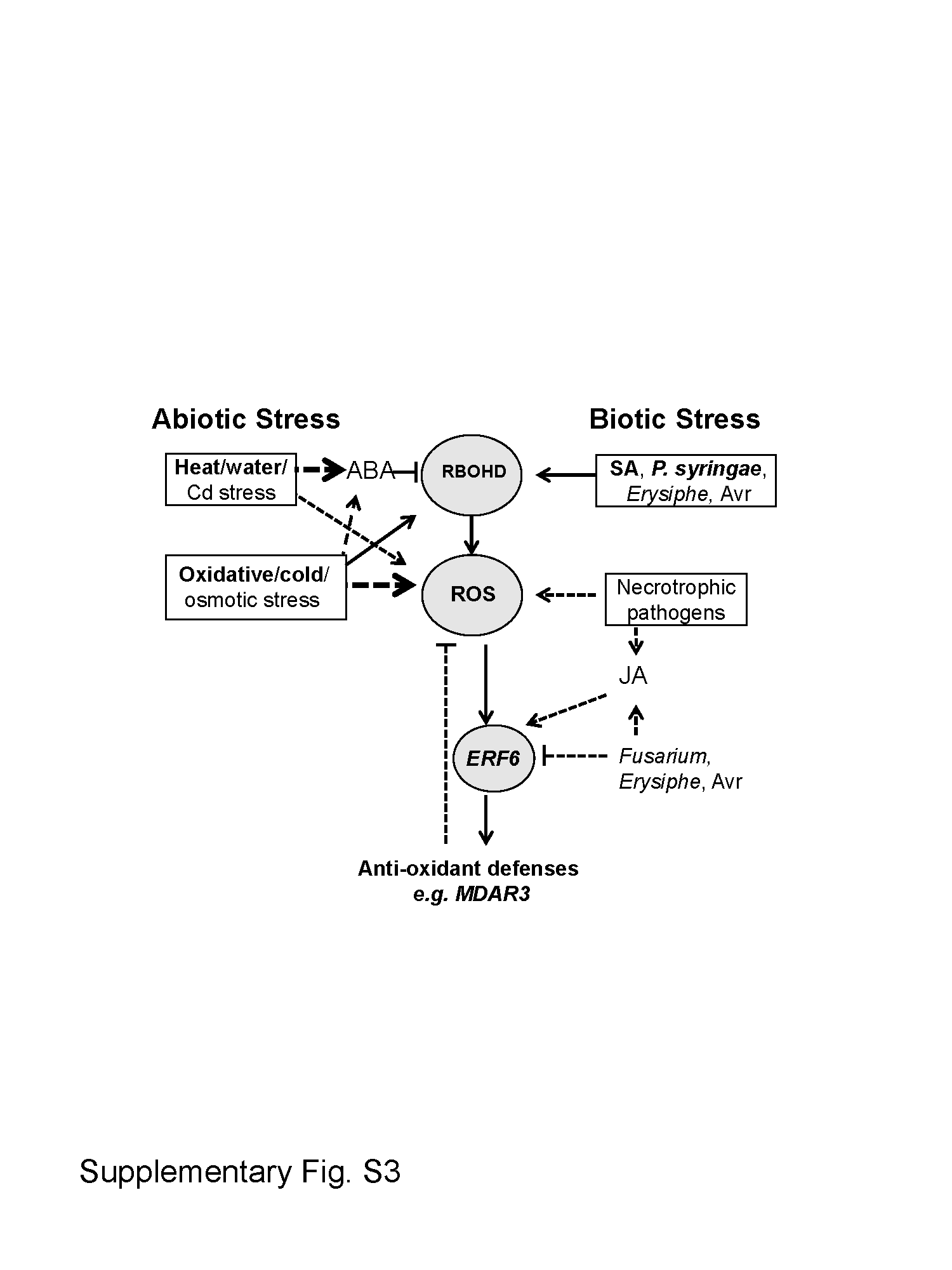

Supplement: Figure S3 — A simplified model proposing the regulatory role of ERF6 in ROS signaling in Arabidopsis. The model combines gene expression data from this study (treatments in bold letters; solid arrows) together with published results [36,37,38,44,64,69,72,76; dashed arrows] and proposes that transcriptional regulation of ERF6 is mostly controlled by ROS levels in plant cells and then leads to a reduction of oxidative stress via anti-oxidant defenses. Cellular ROS levels are influenced by a number of factors, for example various abiotic stresses, NADPH oxidase action and anti-oxidant defenses. Thicker arrows may show the preferred signaling routes of various abiotic stresses that can lead to induction of RbohD and ERF6 for oxidative and cold stress, but suppression by heat and water stress (see Figure 3). In addition, biotic stress caused by successful necrotrophic pathogens may increase ROS levels while typical defense actions against biotrophic pathogens and their elicitors (e.g. Avr) may stimulate ROS production via NADPH oxidase RbohD. Recent experimentation at the protein level has confirmed the role of ERF6 in modulation of cellular oxidative function [72]. (TIF) [file pone.0070289.s003.tif]
